# Use of an Inverse Method for Time Series to Estimate the Dynamics of and Management Strategies for the Box Jellyfish *Carybdea marsupialis*

**DOI:** 10.1371/journal.pone.0137272

**Published:** 2015-09-16

**Authors:** Cesar Bordehore, Verónica L. Fuentes, Jose G. Segarra, Melisa Acevedo, Antonio Canepa, Josep Raventós

**Affiliations:** 1 Department of Ecology and Multidisciplinary Institute for Environmental Studies “Ramon Margalef”, University of Alicante, Alicante, Spain; 2 Institute of Marine Sciences, CSIC, Barcelona, Spain; University of Waikato (National Institute of Water and Atmospheric Research), NEW ZEALAND

## Abstract

Frequently, population ecology of marine organisms uses a descriptive approach in which their sizes and densities are plotted over time. This approach has limited usefulness for design strategies in management or modelling different scenarios. Population projection matrix models are among the most widely used tools in ecology. Unfortunately, for the majority of pelagic marine organisms, it is difficult to mark individuals and follow them over time to determine their vital rates and built a population projection matrix model. Nevertheless, it is possible to get time-series data to calculate size structure and densities of each size, in order to determine the matrix parameters. This approach is known as a “demographic inverse problem” and it is based on quadratic programming methods, but it has rarely been used on aquatic organisms. We used unpublished field data of a population of cubomedusae *Carybdea marsupialis* to construct a population projection matrix model and compare two different management strategies to lower population to values before year 2008 when there was no significant interaction with bathers. Those strategies were by direct removal of medusae and by reducing prey. Our results showed that removal of jellyfish from all size classes was more effective than removing only juveniles or adults. When reducing prey, the highest efficiency to lower the *C*. *marsupialis* population occurred when prey depletion affected prey of all medusae sizes. Our model fit well with the field data and may serve to design an efficient management strategy or build hypothetical scenarios such as removal of individuals or reducing prey. TThis This sdfsdshis method is applicable to other marine or terrestrial species, for which density and population structure over time are available.

## Introduction

Population ecology of marine organisms commonly uses a descriptive approach in which their sizes and densities are plotted over time. This approach is valid for understanding what has occurred in the past, but it has limited usefulness for design strategies in management or modelling to anticipate plausible or theoretical scenarios.

Population projection matrix models are among the most widely used tools in ecology. A robust body of literature has discussed the merits of demographic models, particularly in the context of their utility for management (reviewed in Crone et al. [1]). For the majority of pelagic marine organisms and some terrestrial species, it is difficult or even impossible to mark individuals and follow them over time to determine their vital rates and build a population projection matrix model. Nevertheless, it is possible to sample periodically to calculate population size structure and densities.

Such time-series density data can be used to determine the parameters of a population projection matrix model, as we have done here for the box jellyfish *Carybdea marsupialis* (Linnaeus, 1758). This approach is known as an “inverse problem” [2], unlike the traditional modelling approach, a “forward problem”, that predicts the dynamics from the model and the initial conditions [3]. This inverse approach has rarely been used on aquatic organisms. Two exceptions are Katsanevakis & Verriopoulos [4], who modelled *Octopus vulgaris* in the eastern Mediterranean Sea, and Erwin et al. [5], who modelled the invasion of the aquatic plant *Alternanthera philoxeroides*. To our knowledge, this approach has never been used for jellyfish.

Cubozoans, or box jellyfish, are the smallest class of Cnidaria, with only about 50 species [6], which occur in tropical and subtropical waters. Cubozoans are of great biological and social importance [6] because they are active fish and zooplankton predators [7–9], they have complex eyes and visual capabilities [10], mating behaviour [11], and high to extreme toxicity for humans [12–15]. For example, in Australia some cubozoan species are lethal to humans, provoking severe symptoms and sometimes even death [16]. With respect to the studied species, *C*. *marsupialis*, its sting causes severe pain, burning sensation, erythematous-vesicular eruption and local edema [17–19] and even severe systemic effects, as described for the first time in 2015 [20].

Despite their biological and socioeconomic importance, studies on the population ecology and modelling of cubozoans are scarce [21,22] and none has used a matrix approach. In the closely-related Class Scyphozoa, a descriptive approach of population characteristics over time has been used, e.g. for *Aurelia* sp. [23] and in Class Hydrozoa, e.g. for *Sarsia tubulosa* and *Aequorea vitrina* [24]. Palomares & Pauly [25] reviewed the growth of jellyfishes with the von Bertalanffy formula for scyphozoans and a few cubozoans. In scyphozoans, there are few studies using a matrix approach with size classes [26]. For example, Malej & Malej [27] simulated the abundance of *Pelagia noctiluca* in the northern Adriatic, showing that the most important effect on population density was maturation at an early stage. A matrix approach allows evaluation of the population performance at different life history stages and estimates the possible effects that changes in any of the stages (or ages) may have on the others, thus providing useful information for biological conservation and management [3].*C*. *marsupialis* is the only box jellyfish described in the Mediterranean Sea, where high density areas have been detected along the coast since the 1980s in the Adriatic Sea [28] and since 2008 along Spanish coasts [29]. Boero [30] correlated its distribution to a combination of two main factors: i) the presence of coastal defences–ports and breakwaters to prevent beach erosion, which could be suitable surfaces for polyp settlement, and ii) high abundance of zooplankton prey due to anthropogenic coastal fertilization. In the studied area, *C*. *marsupialis* showed a sudden rise in population abundance in summer 2008 that had never been detected before. This high density has been stable since then and has been responsible for thousands of stings each summer [29].

Thus, the rise of *C*. *marsupialis* in some places in the Mediterranean [20,29,30] is a new problem that managers of coastal waters should address. Our goal was to reduce the jellyfish population, especially the painfully stinging large ones, to improve conditions for tourists. We compared two different approaches, one to directly remove the jellyfish and the other to indirectly reduce their abundance by reducing nutrient inputs, which could be applied to manage this and other species.

To better understand the underlying mechanisms that allow *C*. *marsupialis* to complete its life cycle and maintain a consistently high population density since 2008, we used a matrix model to study and model its population during one medusa-stage season, from mid-2010 to the beginning of 2011.

Our main goals of this paper were to set up a methodology to model marine organisms that cannot be marked and followed, using a population projection matrix model from time-series field density data (inverse problem), And to evaluate two different approaches (jellyfish removal and prey reduction) to reduce *C*. *marsupialis* abundance considering different jellyfish size classes.

## Materials and Methods

### Study area

**Fig 1:**
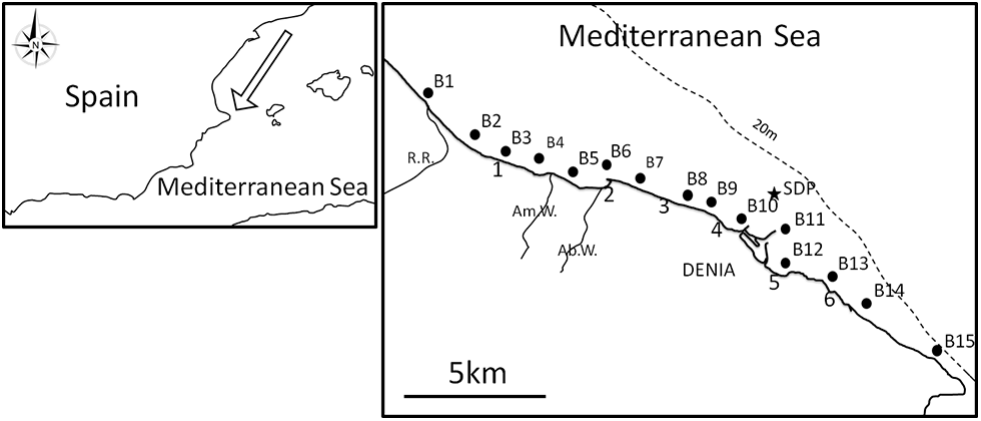
Location of *Carybdea marsupialis* sampling sites. B1 to B15: monthly boat transects, 100 to 200 m from shoreline. 1 to 6: walking transects. See Table 1 for coordinates and beach names. SDP: sewage disposal point (secondary treatment).

The field work was conducted along 17 km of the coast of Denia (SE Spain, W Mediterranean, Fig 1). Among the beaches studied, 1–5 have a gentle slope and the following main substrate types: Almadrava (1), pebbles and sand; Molins (2) and Marines (3), sand; Raset (4), sand and mud; Marineta (5), *Caulerpa prolifera* on mud with some pebbles, and Rotes (6), boulders and pebbles. In deeper waters (~2–4 m depth) of beaches 1–4, the sea bottom is covered by approximately 50% sand and 50% by the seagrass *Posidonia oceanica* at different conservation levels (from regressive to good condition), at beaches 5 and 6 rocky bottoms appear in addition to sand bottoms and *P*. *oceanica* is generally in a better condition.

### Life cycle of *Carybdea marsupialis*

**Fig 2:**
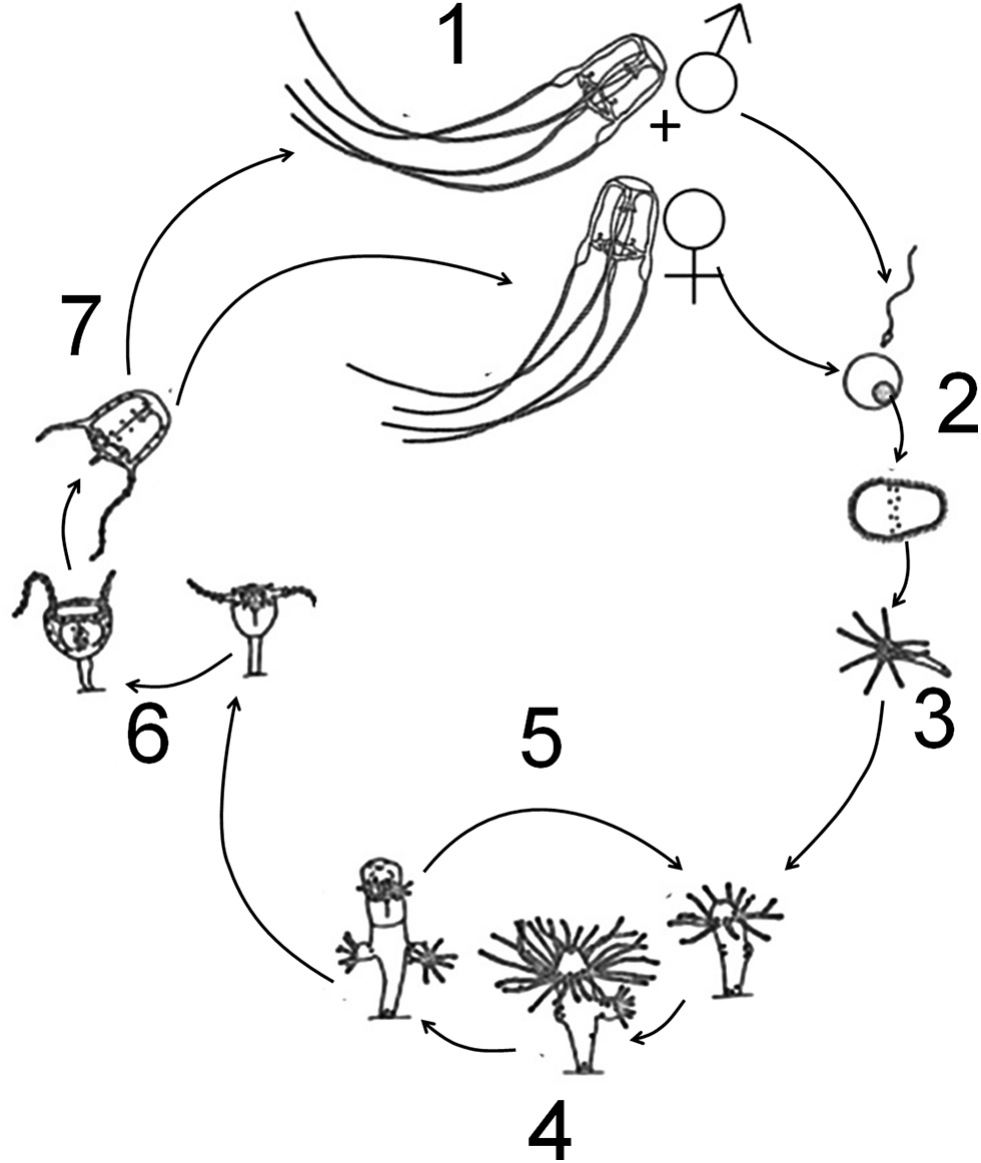
Life cycle of *Carybdea marsupialis*. Male and female mating (1), release of negatively-buoyant fertilized egg into the water column (2), settlement of planula larva on substrate after ~2 days (3), benthic polyp phase (4), new polyps budding from existing polyps (5), polyp metamorphosing into juvenile medusa (6), release juvenile cubomedusa (7). Drawing adapted from Studebaker [31], University of Puerto Rico.

The life cycle of *C*. *marsupialis* (Fig 2) includes male and female mating (1), release of the negatively-buoyant fertilized eggs into the water column (2), settlement of the planula larvae on substrate a few days after ova fertilization [31], Acevedo & Fuentes pers. obs.] (3), the benthic polyp phase (4), new polyps budding from existing polyps (5), mature polyps metamorphosing into juvenile medusae (6), and release of the juvenile cubomedusae (7). *Carybdea marsupialis* medusae are oviparous and dioecious, with males and females having no phenotypic differences except in mature gonads. For the matrix analysis, we consider six stages, in which three of them are juveniles (number 7 in Fig 2) and the other three are adults (number 1 in Fig 2).

### Field measurements

Densities of *Carybdea marsupialis* were quantified by capturing individuals using towed nets in a narrow zone close to the shoreline. A flowmeter was mounted in each net to calculate the water volume filtered. Transects were walked by people with nets at 0.5 to 1.2 m depth, 1 m to ~15 m distant from the shoreline, each tow was walked at a speed of ~1 km h^-1^ along a distance of about 20 to 40 m. We also did periodic boat tows 50 to 100 m from the coast (Fig 1, points B1 to B15), but densities there were very low (<1% total captures) and those densities were not used in the model.

The Spanish Ministry of the Environment (Dirección General de Sostenibilidad de la Costa y el Mar) and the regional environmental authority (Dirección General del Agua) supported LIFE CUBOMED project and authorised samplings. The field studies did not involve endangered or protected species.

Walking tows were done simultaneously with two or three types of nets according to mesh size: 200-μm, 50-cm diameter (375 tows, mean of 3.16 m^3^ filtered per tow); 400-μm, 50-cm diameter (294 tows, mean 5.83 m^3^ filtered); 4-mm 50x50-cm square net (552 tows, mean 12.55 m^3^ filtered). The 200-μm mesh net was used to sample jellyfish smaller than 5 mm diagonal bell width (DBW) measured between opposite pedalia on a flattened specimen, from which we discarded bigger jellyfish (10 from a total of 896), which appeared able to escape from the 200-μm net due to slow water flow. The 400-μm net was used for counts of >400-μm DBW jellyfish. The 4-mm net was used only for those larger than 4 mm DBW, discarding smaller medusae that could pass through the mesh (195 of a total of 2095 jellyfish). Size of each medusa captured was measured (±0.1 mm) using a stereoscopic microscope with graduated ocular for jellyfish ≤5 mm DBW and a calliper for medusae >5 mm DBW.

In May 2010, we only did boat tows at 100–200 m from the shoreline (Fig 1, points B1 to B15), but we captured no jellyfish in 765 m3 of water filtered. The density of medusae in May 2010 for waters close to the shoreline was estimated from walking transects from May 2011. To approximate the medusa population close to the coastline in May 2010, we assumed that (D_May10_/D_June10_) = (D_May11_/D_June11_) and obtained 0.08 ind. m^-3^ for May 2010, which we consider closer to reality than zero.

We made ten attempts from November 2010 to March 2011 to find and quantify the benthic polyps. SCUBA divers scraped hard surfaces and collected small stones and pebbles as well as sand between 0 and 3 m depth, each attempt we collected about 0.5 m^2^ of substratum. The substrate materials were examined with the binocular microscope, but no polyps were found and we were unable to include the polyp stage in our matrix analysis (Fig 2, stage 4). To our knowledge, the polyp stage has not yet been found in the Mediterranean.

For the matrix model we added together samples for all beaches by month. All 6 beaches were sampled each month with a similar sampling effort (Table 1). The matrix was obtained from density data from April 2010 to January 2011.

**Table 1:** Total volume filtered each month (m^3^) and total *Carybdea marsupialis* medusae captured per month near Denia, SE Spain in 2010 and 2011.

	Volume filtered (m^3^)	Medusae captured (numbers)
Jun-10	1030.83	184
Jul-10	631.53	376
Aug-10	927.13	334
Sep-10	3696.16	279
Oct-10	1224.38	134
Nov-10	1101.80	0
Dec-10	343.75	0
Jan-11	877.66	0
Apr-11	126.40	0
May-11	681.13	271
Jun-11	320.88	682
Jul-11	1368.94	913
Oct-11	660.08	0
Total	12864.27	3173

The different classes for the time-series of jellyfish density data (medusae m^-3^) from year 2010 (Table 2) were defined according the following criteria:

- Juvenile 1: 0.2 < DBW < 5 mm. From metamorphosis from the polyp until medusae develop gastric cirri and pedalia, but velar canals are not visible yet.
- Juvenile 2: 5 ≤ DBW < 10 mm. Intermediate between juvenile 1 and juvenile 3. Velar canals are developed.
- Juvenile 3: 10 ≤ DBW < 15 mm. Adult morphology without gonads.
- Adult 1: 15 ≤ DBW < 20 mm. With gonads.
- Adult 2: 20 ≤ DBW < 25 mm. With gonads.
- Adult 3: DBW ≥ 25 mm. With gonads.

**Table 2:** Densities of *Carybdea marsupialis* by month in 2010.

		Apr	May		Jun		Jul		Aug		Sep		Oct		Nov
Stage		Mean	Mean	SE	Mean	SE	Mean	SE	Mean	SE	Mean	SE	Mean	SE	Mean
1	Juvenile 1	0	0.080*	0.027*	0.222	0.050	0.994	0.281	0.462	0.204	0.012	0.007	0.005	0.004	0
2	Juvenile 2	0	0		0.046	0.081	0.048	0.029	0.073	0.031	0.016	0.008	0.001	0.001	0
3	Juvenile 3	0	0		0.001	0.002	0.007	0.004	0.039	0.020	0.023	0.006	0.021	0.008	0
4	Adult 1	0	0		0		0		0.018	0.013	0.028	0.009	0.071	0.035	0
5	Adult 2	0	0		0		0		0.005	0.004	0.018	0.005	0.024	0.015	0
6	Adult 3	0	0		0		0		0		0.005	0.001	0.011	0.005	0

Data in medusae m^-3^.

*Data from May 2010 was estimated (see text for explanation).

### Data arrangement and matrix model

**Fig 3:**
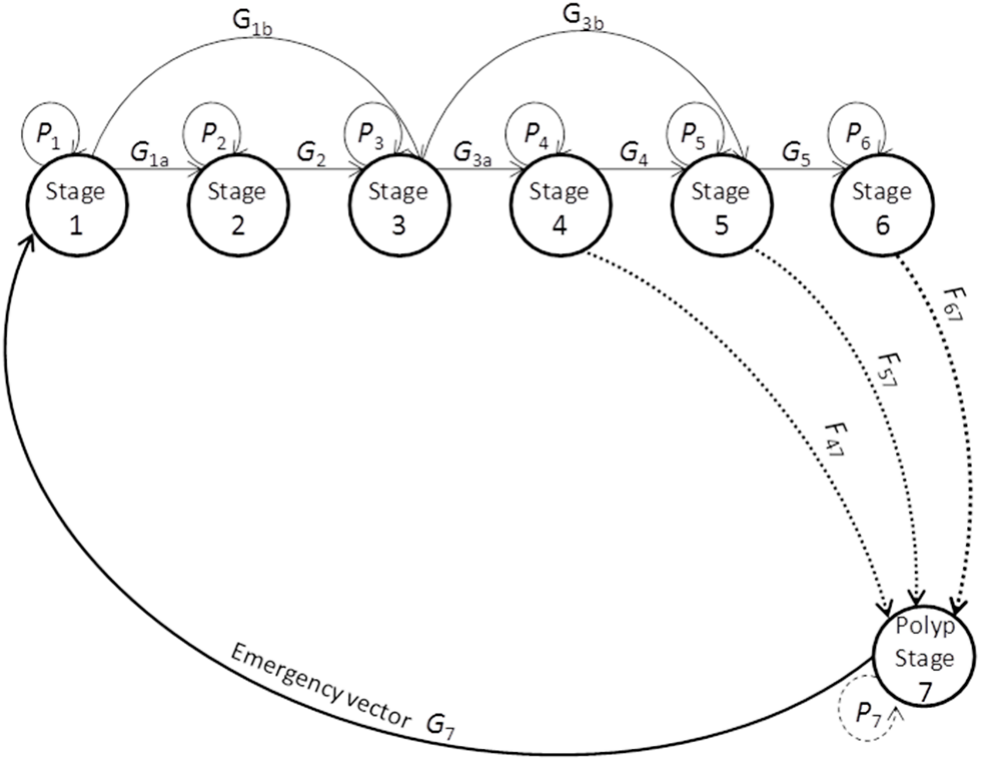
Life stages used in the population projection matrix model of the cubomedusa *Carybdea marsupialis*. *P*_1–7_: individual remains at each stage. *G*_1a–5_: parameter describing growth between stages. *G*_7_: parameter describing new individuals of juvenile cubomedusae from the polyp stage. F: fecundity.

From the life cycle shown in Fig 3, our objective was to develop a population projection matrix with the following structure:

$$A=\left(\begin{array}{cccccc} P_{1} & 0 & 0 & 0 & 0 & 0\\ G_{1a} & P_{2} & 0 & 0 & 0 & 0\\ G_{1b} & G_{2} & P_{3} & 0 & 0 & 0\\ 0 & 0 & G_{3a} & P_{4} & 0 & 0\\ 0 & 0 & G_{3b} & G_{4} & P_{5} & 0\\ 0 & 0 & 0 & 0 & G_{5} & P_{6} \end{array}\right)$$ (1)

Where *P* (= individual remains at that stage) is the parameter describing the cubomedusae not growing into a bigger life stage. *G* (= growth) is the parameter describing growth from an early life stage to a later (bigger) life stage. For example, *G*_1b_ represents the medusae growing from the first stage to the third stage at a time *t*. *G*_7_ is the parameter describing new individuals of juvenile cubomedusae from the polyp stage. The first and third columns of the matrix (Eq 1) have three non-zero elements to accommodate the growth pattern shown in Table 2.

We obtained a non-negative matrix and modelled only the survival and growth processes because, during the eight months of sampling, we did not have accurate reproduction data. Reproductive adults produce planula larvae from October to November (Fuentes & Acevedo pers. obs.), which soon attach to substrata and form polyps. Recently detached juveniles captured in May-June could come from polyps that were in the study area; alternatively, juveniles could come from the north with the general current passing along the coast from North to South, although wind-driven currents can change the currents temporarily. Moreover a polyp can release juveniles the same year that it forms and also in following years by leaving a remnant polyp after strobilation [32]. Therefore, we were unsure if recently detached juveniles captured in May and June came from polyps settled in the same beach months earlier. Therefore, we focused on modelling the dynamics of *C*. *marsupialis* from June to October.

The April distribution in Table 2 corresponds to the initial vector **v(1)**:

$$v(1)=\left(\begin{array}{c} 0\\ 0\\ 0\\ 0\\ 0\\ 0 \end{array}\right)$$ (2)

During the next three months new juvenile 1 were incorporated, as denoted by the symbol **v0**. From August to November there was no incorporation of juvenile 1.

The matrix equation took this form:

$$\begin{array}{cc} v(t+1)=\mathrm{Av}(t)+v0 & t\leq t_{1}\\ v(t+1)=\mathrm{Av}(t) & t>t_{1} \end{array}$$ (3)

where *t*_*1*_ = 4 for the month of July.

The problem was to determine the numerical values of matrix **A** and the emergence vector **v0** from the initial data. To achieve that, we followed the development of Caswell (2001, section 6.2.2) modified by the introduction of the emergence vector **v0**.

First we defined the **M** matrix, corresponding to the data of Table 2 from April to October. Then we defined the vector **z**, corresponding to the data of Table 2 from May to November. After that we generated the matrix of constraints **C** and the vector constraints (**b**). Finally we defined a vector consisting of all the parameters that we wanted to determine (**p**) (S1 File). With all of these elements, we completed specification of the quadratic programming problem, which consisted of:

$$\begin{array}{cc} \text{minimize} & \frac{p^{T}\mathrm{Gp}}{2}+f^{T}p\\ \text{subject to} & \mathrm{Cp}\leq b \end{array}$$

where **G = M^T^M** and **f^T^ = −z^T^M**.

The superscript T on any of the above expressions indicates the transpose of the vector or matrix.

To ensure independence of selection and selective analysis and to deal with and estimate the magnitude of circularity, we compared all data and data split into two sets (odd and even rows, each–representing a plankton net sample) and using odd data to feed the model and even data to compare, and vice versa, as described by Kriegeskorte et al. [33].

### Effectiveness estimation of fishing strategies

To test possible management strategies with our model, we selectively removed different size classes of medusae. Juvenile jellyfish would be eliminated by using tow nets with small mesh size (300-μm) in months before the appearance of adult jellyfish. Adults would be removed by using nets of 15-mm mesh size.

We assumed that in each fishing operation, we would capture a proportion of the existing jellyfish in the study area. We proposed four fishing operations between June to September, which were distributed in different ways. To estimate the effectiveness of each strategy, we calculated the proportion of adult jellyfish, which inflict painful stings, that were not captured in the three months from July to September (bathing months). The proportion of uncaptured jellyfish in a month, after the **n** fishing operations within that month was calculated by the formula **(1-x)** ^**n**^, where **x** is the proportion of jellyfish captured. The number of adult jellyfish not captured in that month was obtained by multiplying the previous proportion by the number of adult jellyfish, which was obtained from vector **v(t)** of equation (Eq 3). Finally, the proportion of adult jellyfish not captured in the three months was calculated by dividing the sum of the number of adult jellyfish not captured in those months by the sum of the number of adult jellyfish in the same months when there were no fishing operations.

### Diminishing available prey for *C*. *marsupialis*

To explore this strategy with our model, we hypothesised that we could reduce prey density, e.g. by reducing the main source of anthropogenic nutrients in the region that come from continental inputs, including agriculture, a diffuse source, and waste water, a point source.

We explored two theoretical scenarios: first, that a reduction of 50% of nutrient input causes a 50% reduction in the jellyfish population parameters by reducing prey densities, and second, that a reduction of nutrients by 50% causes a 25% reduction in the population parameters

The Racons River releases 69.9·10^6^ m^3^ y^-1^ superficially and 40.5·10^6^ m^3^ y^-1^ from aquifer submarine discharge. These high volumes come from the Marjal Pego-Oliva Natural Park, a Ramsar wetland just 1.5 km inland. In the southern part of our study area the aquifer discharge is ~7·10^6^ m^3^ y^-1^. This water is rich both in N and P due to intensive agriculture with 5 to 20 mg N_t_ l^-1^ for the Racons area [34] and 17 to 32 mg N_t_ l^-1^ from the southern aquifer [35]. Concentrations of P_t_ in continental waters (rivers and aquifer) vary from 0.19 to 0.29 mg l^-1^ [36].

The Denia-Ondara-Pedreguer sewage treatment plant releases about 7·10^6^ m^3^ y^-1^. N and P concentrations are high due to the lack of N+P reduction treatment, with mean values of 50–80 mg l^-1^ N_t_, 7–15 P_t_ mg l^-1^ [37]. The mean concentrations allowed us to calculate the following amounts released: N_t_ 1941 mt y^-1^ (79.9% from agriculture and 20.1% from waste water), and P_t_ 88 t y^-1^ (25.1% from agriculture and 74.9% from waste water).

We assumed that if nutrient inputs were lower, phytoplankton densities would be lower as well, and thus zooplankton. Consequently, population parameters of the matrix equation (Eq 3) would be affected.

## Results

For our first objective, we obtained the matrix

$$\text{A}=\left(\begin{array}{cccccc} 0.429 & 0 & 0 & 0 & 0 & 0\\ 0.074 & 0.007 & 0 & 0 & 0 & 0\\ 0.034 & 0.042 & 0.172 & 0 & 0 & 0\\ 0 & 0 & 0.584 & 1 & 0 & 0\\ 0 & 0 & 0.244 & 0 & 0.842 & 0\\ 0 & 0 & 0 & 0 & 0.158 & 1 \end{array}\right)$$ (4)

With the following emergence vector

$$v0={\left(\begin{array}{cccccc} 0.543\; & 0\; & 0\; & 0\; & 0\; & 0 \end{array}\right)}^{T}$$ (5)

**Fig 4:**
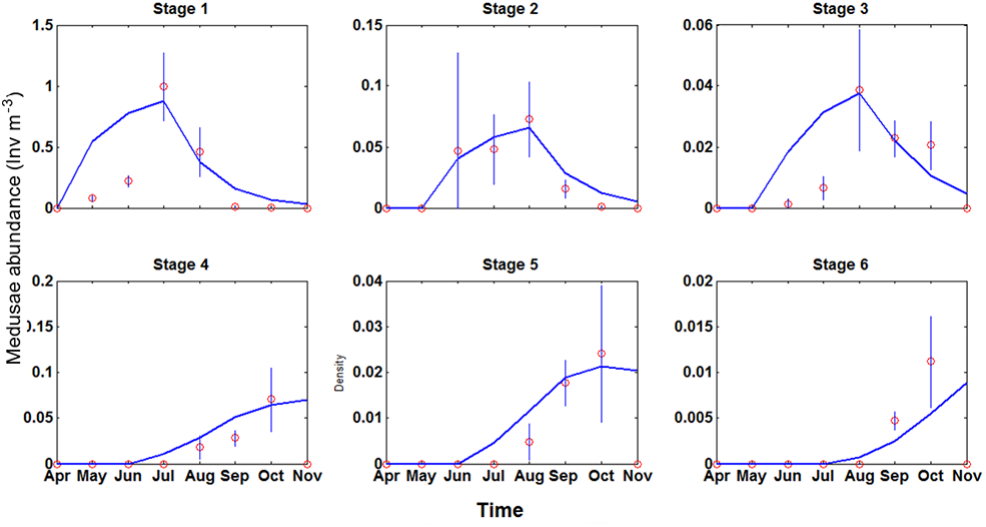
Comparison between abundance data from the field (circles) and calculated with the matrix model (line) for the six stages of growth of *Carybdea marsupialis* jellyfish.

Captured medusae totalled 1207 in 2010 and 1866 in 2011. Table 1 shows the filtered volume and captures by month. Table 2 shows densities of *Carybdea marsupialis* medusae by month in 2010. Those data were compared with the output generated by equation (Eq 3), matrix (Eq 4) and vector (Eq 5) in Fig 4. In the six levels studied, the Chi-square test gave no significant differences between the observed and expected values at a significance level of 95%: level 1: 1.024; level 2: 0.019; level 3: 0.046; level 4: 0.026; level 5: 0.009; level 6: 0.008. The degrees of freedom was 8–1 = 7. Because the values of all levels were below $\chi_{0.01}^{2}$ = 1.24, it indicated that the agreement between the theoretical and the measured data was acceptable.

**Fig 5:**
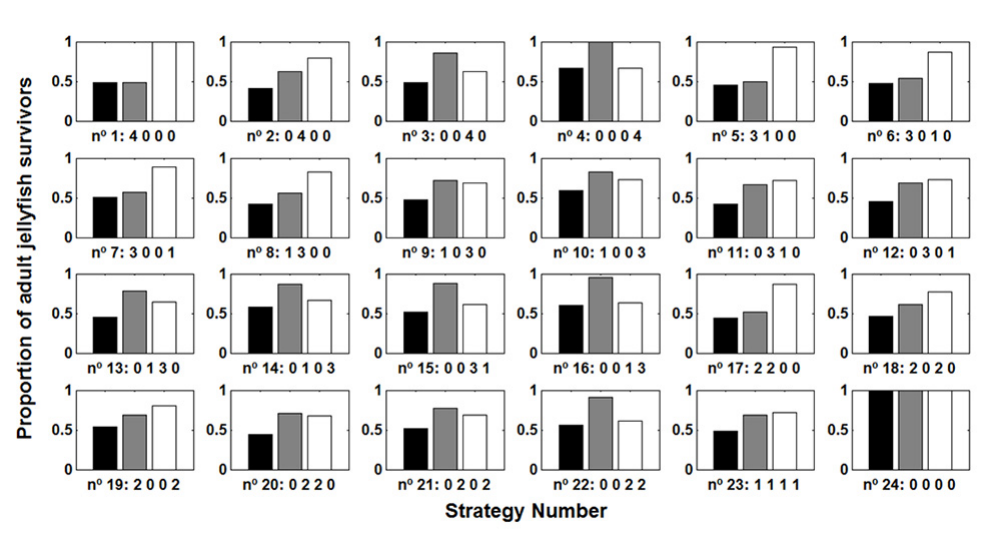
Reduction of the population of adult *Carybdea marsupialis* by application of different removal strategies. For each fishing strategy there are three bars. The left bar (black) capturing jellyfish of all sizes; the middle bar (grey) capturing juveniles; the right bar (white) capturing only adult jellyfish. Each strategy consisted of four removal operations during June to September. The code indicates the number of removal operations each month, e.g. strategy 1 (4 0 0 0) consisted of four removals in June; strategy 6 (3 0 1 0) consisted of three removals in June and one in August. For example, x = 0.2, means that each removal will capture 80% of the jellyfish.

Our second objective was to model the population dynamics of *C*. *marsupialis* to find efficient strategies to lower the population to densities before 2008 when fewer people were stung during summer [29]. In general, removal of jellyfish from all size classes (first bar of each trio, Fig 5) was more effective than removing only juveniles (second bar) or adults (third bar). This is possibly because reduction in the number of individuals was greater when the same proportion of individuals was captured from all classes than when the same proportion of individuals was captured from only 1 or 2 classes.

**Fig 6:**
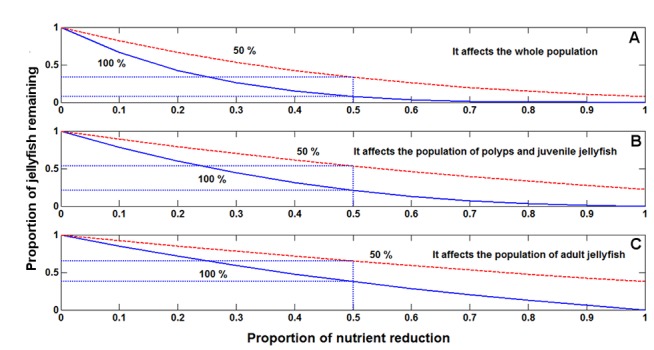
Modelling *Carybdea marsupialis* scenarios of nutrient-prey reduction. Modelling results of the scenario assuming that the reduction of continental nutrient input affects the polyp and medusa stages of *Carybdea marsupialis* by reducing prey density (A). Scenario assuming that nutrient input reduction only affects prey of polyps and juvenile jellyfish (B). Scenario assuming that nutrient input reduction only affects prey of adult jellyfish (C).

The proportion of adult jellyfish was reduced more when prey depletion affected the entire jellyfish population (Fig 6A). When nutrient depletion affected juveniles and polyps (Fig 6B), reduction of adult jellyfish was greater than when it affected only the adult population (Fig 6C).

The model predicted that the three classes of juvenile jellyfish disappeared in the autumn, which agreed with the field measurements and life cycle of the species (Fig 4). Conversely, the model predicted that the three adult classes were present during autumn, even though field measurements showed that they disappeared from the area at the beginning of November. When we forced the model to fit field data during autumn, the goodness of fit of the model during summer was not as strong. Because this paper focused on the dynamics of jellyfish during summer, we chose the strategy that did not allow adults to disappear.

The most effective strategies to reduce the population of adult jellyfish were 2, 8, 11, 17 and 20, assuming removal of all sizes (Fig 5, black bars). With the exception of the number 20, all these strategies focused fishing efforts in July, with high densities of juveniles and adults began to increase. If we considered only juvenile removal (grey bars), strategies 1 and 5 were most effective, which focused on June when juveniles were very abundant. If we considered only adult jellyfish removal (white bars), strategies 15, 16 and 22 were most effective, focusing on the second half of the summer when the adult population was more abundant. In general, jellyfish removal from all classes (black bars) was more effective than only removing juveniles (grey bars) or adults (white bars).

MATLAB programs created for this paper can be downloaded in S2 to S5 Files (S2 File, S3 File, S3 File and S5 File). In S6 File we show how we estimated the effect of double dipping on the validity of the model. This shows that the effect of circularity is negligible because distortions due to double dipping were small. We opted to use all observed data to feed the model and calculate the A matrix, because considering the great variability of the field data (see Figures A to F in S6 File), the more data we use, the more likely the model will represent the real abundance of *C*. *marsupialis*.

## Discussion

The matrix we obtained (Eq 4) from densities and sizes of *C*. *marsupialis* medusae over time allowed us to manipulate different population parameters to model the species’ responses to different scenarios.

Although the model incorrectly predicted the presence of adults in autumn because jellyfish grew into adults later than juveniles, when we forced the model to fit field data during autumn, the goodness of fit of the model during summer was worse. Because this paper focused on the dynamics of jellyfish during summer, we chose the strategy that did not allow adults to disappear.

This matrix approach would allow modelling of different scenarios such as: *a)* management of invasive species, e.g. what size of an invasive species should be targeted for removal in order to find a natural predator or start large-scale removal; *b)* management of bottom-up effects, e.g. what effect would have reduction of prey availability?

### Strategies: depletion vs prey reduction

Figs 5 and 6 suggested that the most effective strategy was to remove jellyfish from all size classes. Alternatively, if removal were limited to only a few classes, it would be more effective to target the juveniles rather than the adults. The best strategies that focused only on juveniles (1 and 5) were more effective than the best strategies that were based on only adults (15, 16, 22); thus, only adult removal was usually less effective than only juvenile removal.

The results by reducing prey paralelled the overall conclusion of jellyfish removal (Fig 5); specifically, the most effective strategy was when the whole population was affected and that removing only adults was generally less effective than removing only juveniles.

The continuous curve in Fig 6 (labeled 100%) was obtained by applying our first assumption, a reduction of 50% of continental nutrient input caused a 50% reduction in the population parameters of the entries of matrix (Eq 4). This scenario could apply to oligotrophic seawaters. The dashed line (labeled 50%) was obtained under the second scenario, where a reduction of continental nutrient inputs by 50% caused a 25% reduction in the population parameters. We do not claim that this is the real situation, because changes in nutrient levels have a complex effects on phytoplankton and zooplankton composition and biomass [38,39]. Nevertheless, because *C*. *marsupialis* feeds mainly on mesozooplanktonic crustaceans [40], we believe our hypothesis is plausible. This effect of bottom-up ecosystem control due to nutrients from agriculture was also described in Mar Menor, a semi-enclosed saline coastal lagoon in SE Spain where increased nutrient discharge has considerably increased jellyfish populations since 1998 [41]. Although we worked on a theoretical scenario, future studies could quantify the relationships between nutrient concentrations, densities of prey and densities of *C*. *marsupialis*, and whether it is feasible to reduce anthropogenic nutrient inputs.

Reducing prey (Fig 6) was more effective than the capture of jellyfish (Fig 5) because growth rates were reduced during all growth processes; meanwhile capture of jellyfish affected only the densities of certain sizes classes during four isolated fishing events (Fig 5).

TThis This sdfsdshis study can provide insight into the dynamics of other species from which there are populations data over time through modelling virtual possible scenarios. Our next step is to produce a more realistic approach by incorporating a sensitivity analysis of the advection and diffusion characteristics of each stage (juveniles and adults) into the present matrix, following the methods developed by Neubert & Caswell [42].

## Supporting Information

S1 File Calculation of the vector containing all model parameters of the box jellyfish *Carybdea marsupialis*. (DOCX)

S2 File Nout7c. Database necessary to execute MATLAB program cubomedusa1.
(MAT)

S3 File Cubomedusa1. MATLAB program that generates a matrix equation that gives the best fit to the data obtained by sampling. It also plots Fig 4.
(M) Click here for additional data file.

S4 File Cubomedusa2. MATLAB program that determines the best strategy to reduce jellyfish population by removal of different size classes. It also plots Fig 5.
(M) Click here for additional data file.

S5 File Cubomedusa3. MATLAB program that determines the best strategy to reduce jellyfish population by decreasing prey or nutrients. It also plots Fig 6.
(M) Click here for additional data file.

S6 File Dealing with circularity. In all figures, black lines show monthly means for observed abundances from *all*, *odd*, and *even* data. Error intervals refer to 95% confidence intervals for *all* data. Solid color lines show the estimated abundances using *odd*, *even*, or *all* data. Class 1 **(Figure A).** Class 2 **(Figure B)**. Class 3 **(Figure C)**. Class 4 **(Figure D)**. Class 5 **(Figure E)**. Class 6 **(Figure F)**.
(DOCX)
