# Supplementary material for: Use of an Inverse Method for Time Series to Estimate the Dynamics of and Management Strategies for the Box Jellyfish Carybdea marsupialis
Source: PLoS One. 2015 Sep 16;10(9):e0137272. doi: 10.1371/journal.pone.0137272 (PMC4573988; doi:10.1371/journal.pone.0137272)
Supplement: S1 File — (DOCX) [file pone.0137272.s001.docx]

**S1 File. Calculation of the vector containing all model parameters of**  **the box jellyfish *Carybdea marsupialis***

First, we define the **M** matrix, corresponding to the data of Table 2 from April to October:

0 0 0 0 0 0 0 0 0 0 0 0 0 0

0 0 0 0 0 0 0 0 0 0 0 0 0 0

0 0 0 0 0 0 0 0 0 0 0 0 0 0

0 0 0 0 0 0 0 0 0 0 0 0 0 0

0 0 0 0 0 0 0 0 0 0 0 0 0 0

0 0 0 0 0 0 0 0 0 0 0 0 0 0

0.080 0 0 0 0 0 0 0 0 0 0 0 0 1.000

0 0.080 0 0 0 0 0 0 0 0 0 0 0 0

0 0 0.080 0 0 0 0 0 0 0 0 0 0 0

0 0 0 0 0 0 0 0 0 0 0 0 0 0

0 0 0 0 0 0 0 0 0 0 0 0 0 0

0 0 0 0 0 0 0 0 0 0 0 0 0 0

0.222 0 0 0 0 0 0 0 0 0 0 0 0 1.000

0 0.222 0 0.047 0 0 0 0 0 0 0 0 0 0

0 0 0.222 0 0.047 0.001 0 0 0 0 0 0 0 0

0 0 0 0 0 0 0.001 0 0 0 0 0 0 0

0 0 0 0 0 0 0 0.001 0 0 0 0 0 0

**M**= 0 0 0 0 0 0 0 0 0 0 0 0 0 0

0.994 0 0 0 0 0 0 0 0 0 0 0 0 0

0 0.994 0 0.048 0 0 0 0 0 0 0 0 0 0

0 0 0.994 0 0.048 0.007 0 0 0 0 0 0 0 0

0 0 0 0 0 0 0.007 0 0 0 0 0 0 0

0 0 0 0 0 0 0 0.007 0 0 0 0 0 0

0 0 0 0 0 0 0 0 0 0 0 0 0 0

0.462 0 0 0 0 0 0 0 0 0 0 0 0 0

0 0.462 0 0.073 0 0 0 0 0 0 0 0 0 0

0 0 0.462 0 0.073 0.039 0 0 0 0 0 0 0 0

0 0 0 0 0 0 0.039 0 0.018 0 0 0 0 0

0 0 0 0 0 0 0 0.039 0 0.018 0.005 0 0 0

0 0 0 0 0 0 0 0 0 0 0 0.005 0 0

0.012 0 0 0 0 0 0 0 0 0 0 0 0 0

0 0.012 0 0.016 0 0 0 0 0 0 0 0 0 0

0 0 0.012 0 0.016 0.023 0 0 0 0 0 0 0 0

0 0 0 0 0 0 0.023 0 0.028 0 0 0 0 0

0 0 0 0 0 0 0 0.023 0 0.028 0.018 0 0 0

0 0 0 0 0 0 0 0 0 0 0 0.018 0.005 0

The last column of the matrix M corresponds to the vector of emergence. This column has two entries with value 1, corresponding to the emergence value of the months of May and June. Although there should be another 1 at the top of the far-right column, corresponding to the emergence value of April, when we placed that 1, the mean quadratic error was lower (rms = 0.0144), but then the density of jellyfish was underestimated in the months when it was higher (July and August). By deleting that 1, the general adjustment was worse (rms = 0.0181), but the maximum density of jellyfish was better estimated. Because our intention was to use the model to design strategies to reduce the plague of jellyfish in the months of their highest densities, we chose the second option.

Then we defined the vector **z**, corresponding to the data of Table 1 from May to November:

**z**=(0.080 0 0 0 0 0 0.222 0.046 0.001 0 0 0.994 0.048 0.007 0 0 0 0.462 0.073 0.039 0.018 0.005 0 0.012 0.016 0.023 0.028 0.018 0.005 0.005 0.001 0.021 0.071 0.024 0.011)

Then we generated the matrix of constrains:

$$\mathbf{C}=\left(\begin{matrix} -1 & 0 & 0 & 0 & 0 & 0 & 0 & 0 & 0 & 0 & 0 & 0 & 0 & 0 \\ 0 & -1 & 0 & 0 & 0 & 0 & 0 & 0 & 0 & 0 & 0 & 0 & 0 & 0 \\ 0 & 0 & -1 & 0 & 0 & 0 & 0 & 0 & 0 & 0 & 0 & 0 & 0 & 0 \\ 0 & 0 & 0 & -1 & 0 & 0 & 0 & 0 & 0 & 0 & 0 & 0 & 0 & 0 \\ 0 & 0 & 0 & 0 & -1 & 0 & 0 & 0 & 0 & 0 & 0 & 0 & 0 & 0 \\ 0 & 0 & 0 & 0 & 0 & -1 & 0 & 0 & 0 & 0 & 0 & 0 & 0 & 0 \\ 0 & 0 & 0 & 0 & 0 & 0 & -1 & 0 & 0 & 0 & 0 & 0 & 0 & 0 \\ 0 & 0 & 0 & 0 & 0 & 0 & 0 & -1 & 0 & 0 & 0 & 0 & 0 & 0 \\ 0 & 0 & 0 & 0 & 0 & 0 & 0 & 0 & -1 & 0 & 0 & 0 & 0 & 0 \\ 0 & 0 & 0 & 0 & 0 & 0 & 0 & 0 & 0 & -1 & 0 & 0 & 0 & 0 \\ 0 & 0 & 0 & 0 & 0 & 0 & 0 & 0 & 0 & 0 & -1 & 0 & 0 & 0 \\ 0 & 0 & 0 & 0 & 0 & 0 & 0 & 0 & 0 & 0 & 0 & -1 & 0 & 0 \\ 0 & 0 & 0 & 0 & 0 & 0 & 0 & 0 & 0 & 0 & 0 & 0 & -1 & 0 \\ 0 & 0 & 0 & 0 & 0 & 0 & 0 & 0 & 0 & 0 & 0 & 0 & 0 & -1 \\ 1 & 1 & 1 & 0 & 0 & 0 & 0 & 0 & 0 & 0 & 0 & 0 & 0 & 0 \\ 0 & 0 & 0 & 1 & 1 & 0 & 0 & 0 & 0 & 0 & 0 & 0 & 0 & 0 \\ 0 & 0 & 0 & 0 & 0 & 1 & 1 & 1 & 0 & 0 & 0 & 0 & 0 & 0 \\ 0 & 0 & 0 & 0 & 0 & 0 & 0 & 0 & 1 & 1 & 0 & 0 & 0 & 0 \\ 0 & 0 & 0 & 0 & 0 & 0 & 0 & 0 & 0 & 0 & 1 & 1 & 0 & 0 \\ 0 & 0 & 0 & 0 & 0 & 0 & 0 & 0 & 0 & 0 & 0 & 0 & 1 & 0 \end{matrix}\right)$$

Then we generated the vector constrains:

$$\mathbf{b}=\left(\begin{matrix} 0 & 0 & 0 & 0 & 0 & 0 & 0 & 0 & 0 & 0 & 0 & 0 & 0 & 0 & 1 & 1 & 1 & 1 & 1 & 1 \end{matrix}\right)$$

Finally we defined a vector consisting of all the parameters that we wanted to determine:

$$\mathbf{p=}\left(\begin{matrix} P_{1} & G_{1a} & G_{1a} & P_{2} & G_{2} & P_{3} & G_{3a} & G_{3b} & P_{4} & G_{4} & P_{5} & G_{5} & P_{6} & v0 \end{matrix}\right)$$
