# Supplementary material for: Use of an Inverse Method for Time Series to Estimate the Dynamics of and Management Strategies for the Box Jellyfish Carybdea marsupialis
Source: PLoS One. 2015 Sep 16;10(9):e0137272. doi: 10.1371/journal.pone.0137272 (PMC4573988; doi:10.1371/journal.pone.0137272)
Supplement: S6 File — In all figures, black lines show monthly means for observed abundances from all, odd, and even data. Error intervals refer to 95% confidence intervals for all data. Solid color lines show the estimated abundances using odd, even, or all data. Class 1 (Figure A). Class 2 (Figure B). Class 3 (Figure C). Class 4 (Figure D). Class 5 (Figure E). Class 6 (Figure F). (DOCX) [file pone.0137272.s006.docx]

**S6. Dealing with circularity**

To avoid circularity in the model, which is using the same set of data to feed the model and to test the model, and to ensure the independence of selection and selective analysis, we split the original data into two sets of equal number of samples (odd and even rows, each row being one sample -a plankton net tow-) following Kriegeskort et al. [1].

Once the data were split, we used the *odd* group to estimate the parameters of the matrix, as explained in Materials and Methods, and the *even* data group to test whether the results of the model (expected values) fit the observed data. We also did the reverse. We also maintained the original model (using all captures as observed data). In addition, we compared the “all data” abundance with the odd and even abundances to test if this split into two halves significantly changed the observed abundance values.

As shown in Figures A to F (Figure A in S6 File, Figure B in S6 File, Figure C in S6 File, Figure D in S6 File, Figure E in S6 File, Figure F in S6 File) within each size class, the *odd* and *even* abundance data are contained within the 95% confidence intervals of *all* data, showing the same temporal pattern, with the only exception for June Class 2, where *odd* and *even* differed greatly from *all* because medusa captures were found only in one sample (plankton net).Thus, by chance, all the captures were assigned to *odd* and none to *even*. Despite this effect, the zero value for *even* June Class 2 is contained between *all* 95% confidence intervals.

No matter which data we used as observed values (*all*, *odd* or *even*), the model offered a satisfactory result (solid lines in Figures A to F). Even though the estimated abundances for May and June in Class 1 (Figure A) and June and July for Class 3 (Figure C), were overestimated, the effects on the validity of the matrix model under the different management scenarios (diminish prey, capture medusae) shown in the manuscript are nil because we only account for the population of adults (Classes 4 to 6), which are the sizes that inflict stings on humans.

Because we saw that the matrix model was able to model the population of *Carybdea marsupialis* and that the effects of circularity were negligible [1], we believe that considering the great variability of field data, the more data we use, then the more accurate the model would be; therefore, we should use all of the field data to feed our model and predict what changes will occur to the population under the different hypotheses.


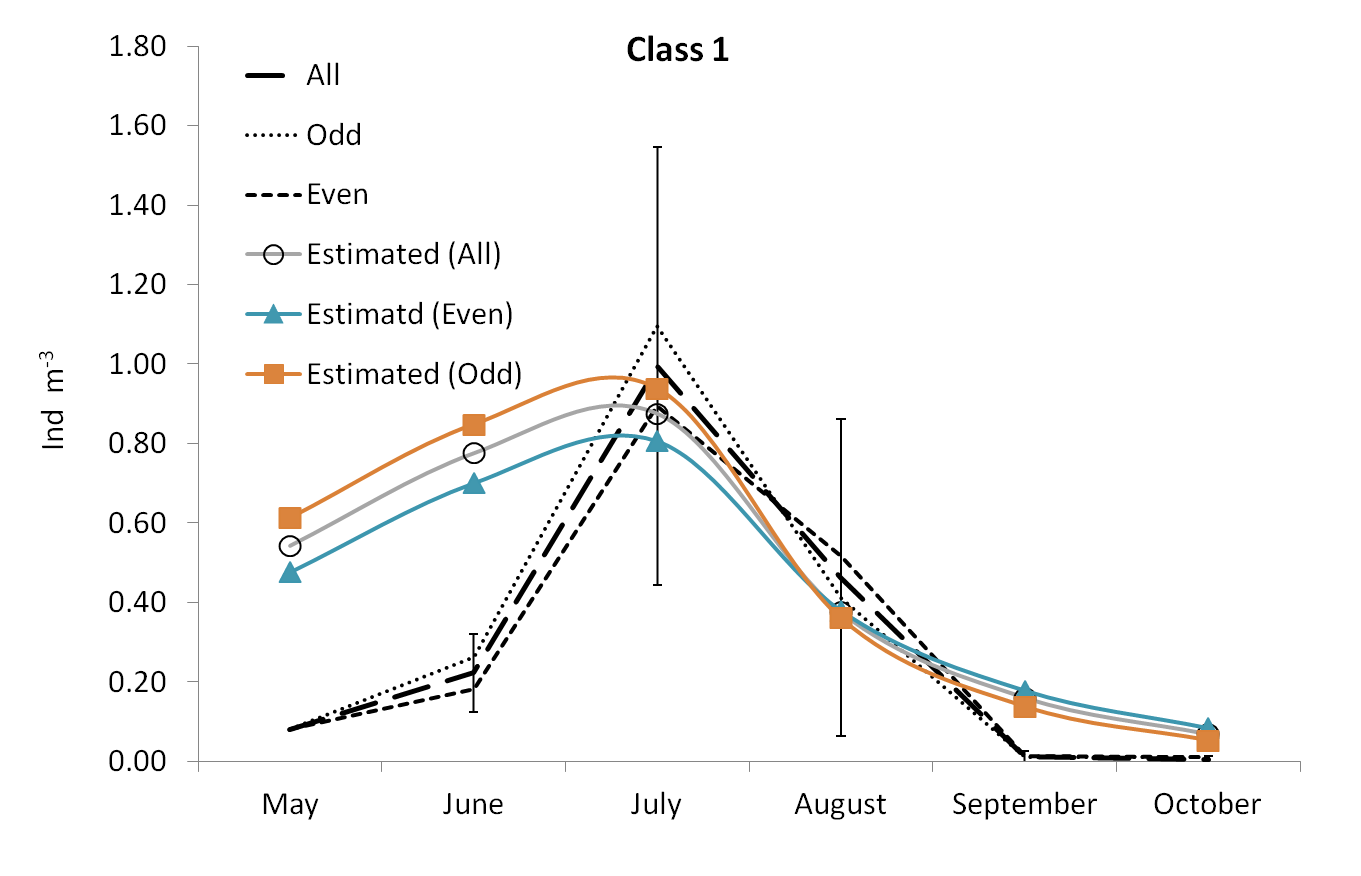


**Figure A. Class 1.** Black lines show monthly means for observed abundances from *all*, *odd*, and *even* data. Error intervals refer to 95% confidence intervals for *all* data. Solid color lines show the estimated abundances using *odd*, *even*, or *all* data.


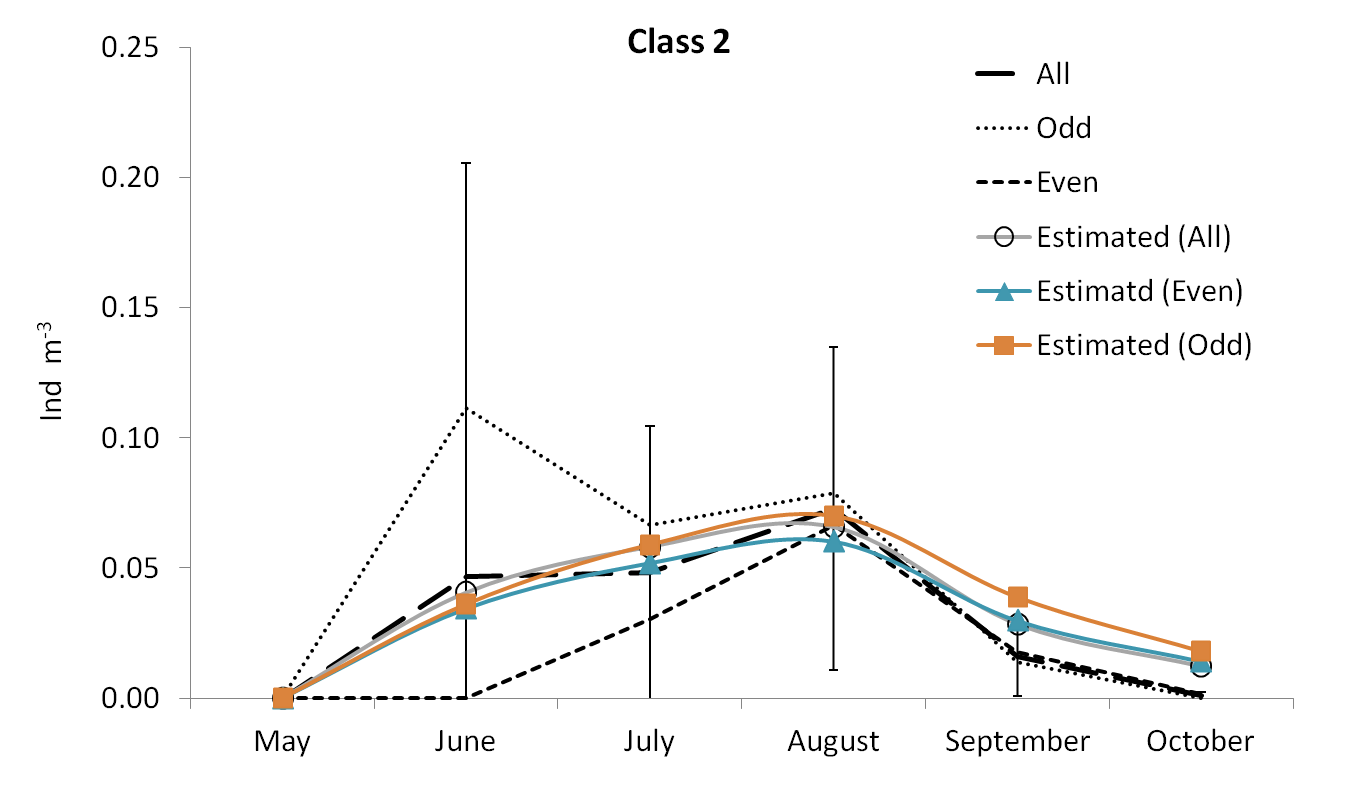


**Figure B. Class 2.** Black lines show monthly means for observed abundances from *all*, *odd*, and *even* data. Error intervals refer to 95% confidence intervals for *all* data. Solid color lines show the estimated abundances using *odd*, *even*, or *all* data.


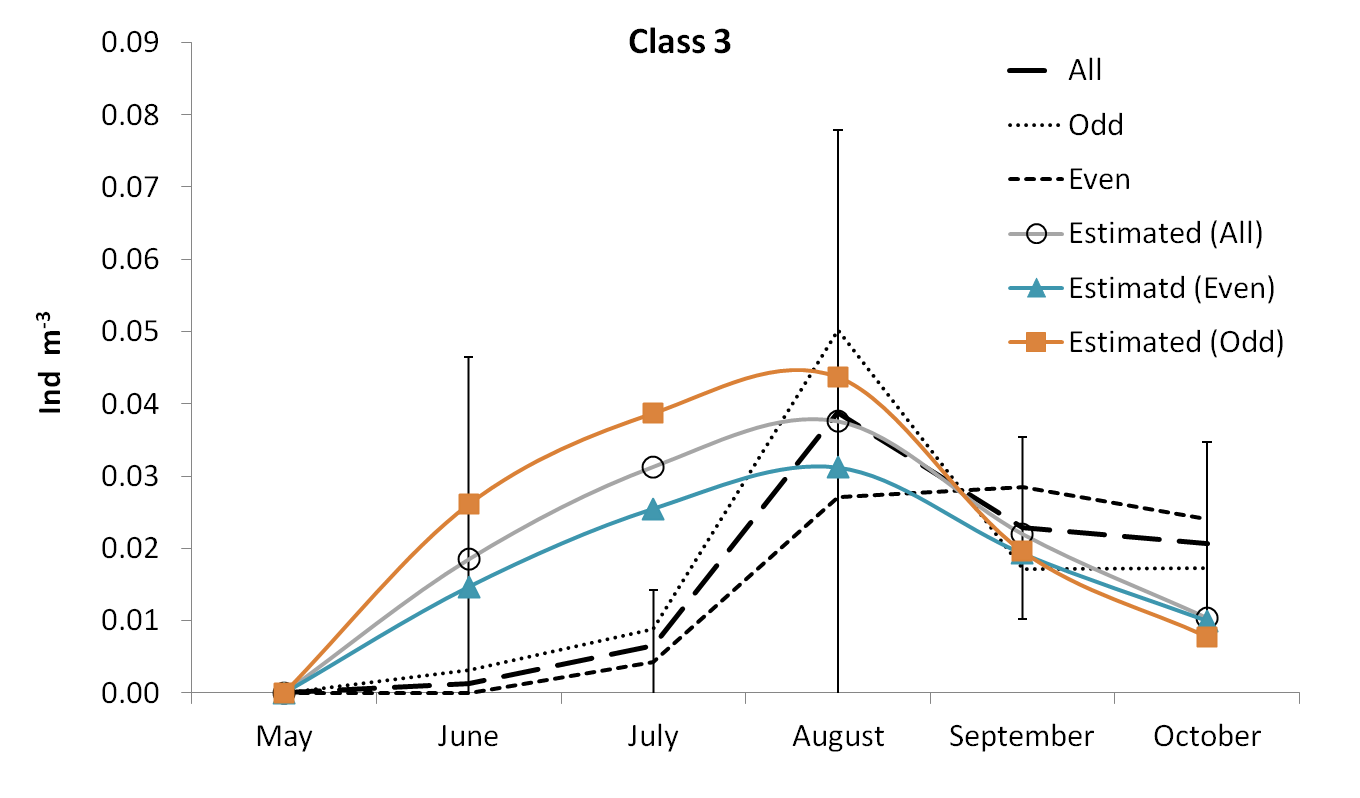


**Figure C. Class 3.** Black lines show monthly means for observed abundances from *all*, *odd*, and *even* data. Error intervals refer to 95% confidence intervals for *all* data. Solid color lines show the estimated abundances using *odd*, *even*, or *all* data.


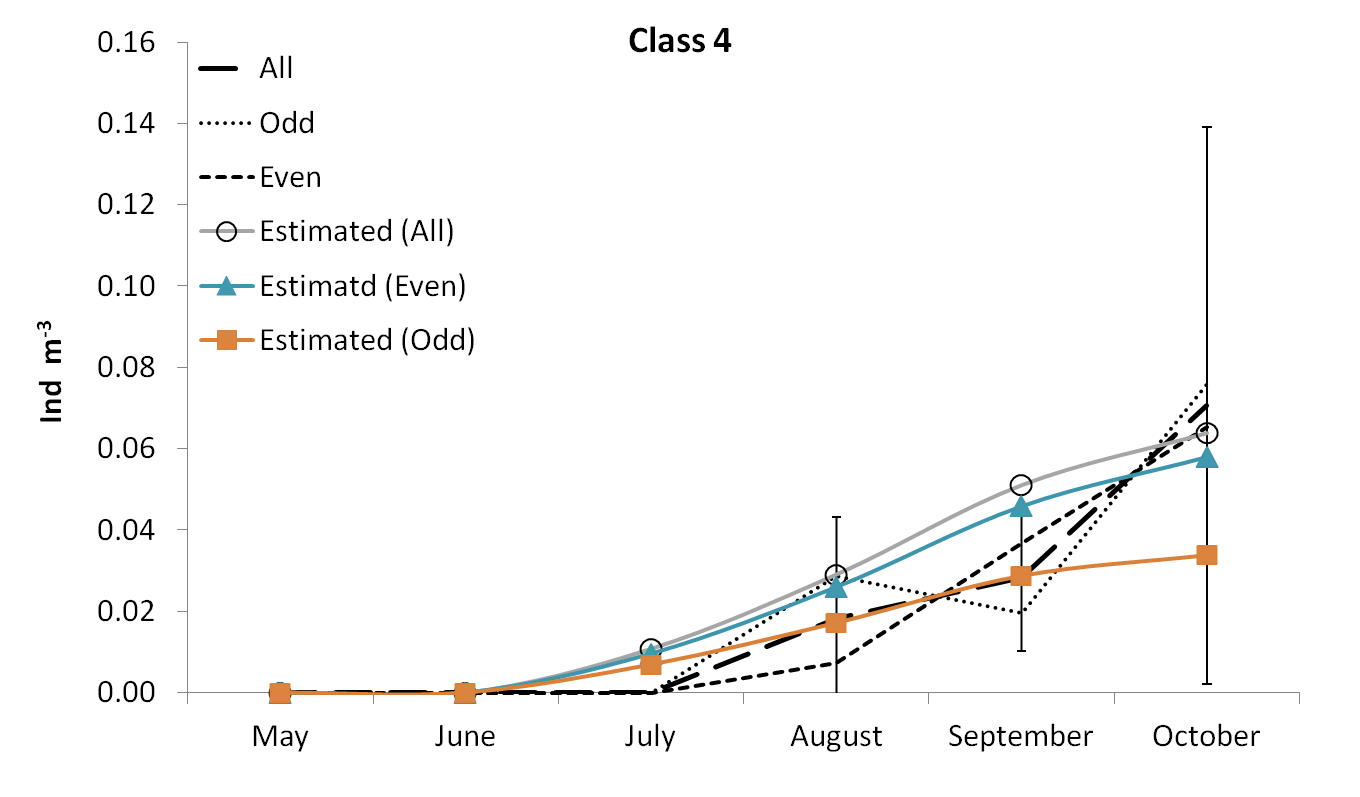


**Figure D. Class 4.** Black lines show monthly means for observed abundances from *all*, *odd*, and *even* data. Error intervals refer to 95% confidence intervals for *all* data. Solid color lines show the estimated abundances using *odd*, *even*, or *all* data.


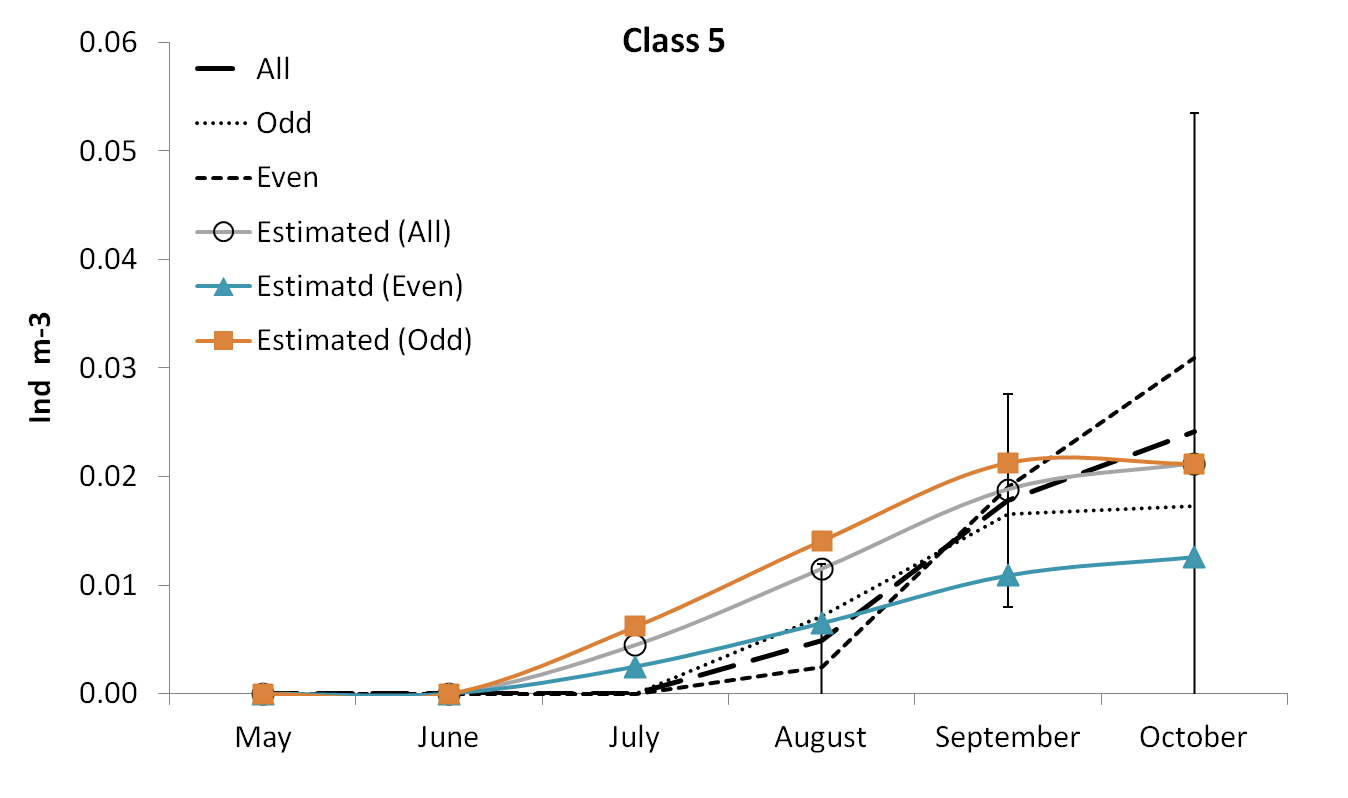


**Figure E. Class 5.** Black lines show monthly means for observed abundances from *all*, *odd*, and *even* data. Error intervals refer to 95% confidence intervals for *all* data. Solid color lines show the estimated abundances using *odd*, *even*, or *all* data.


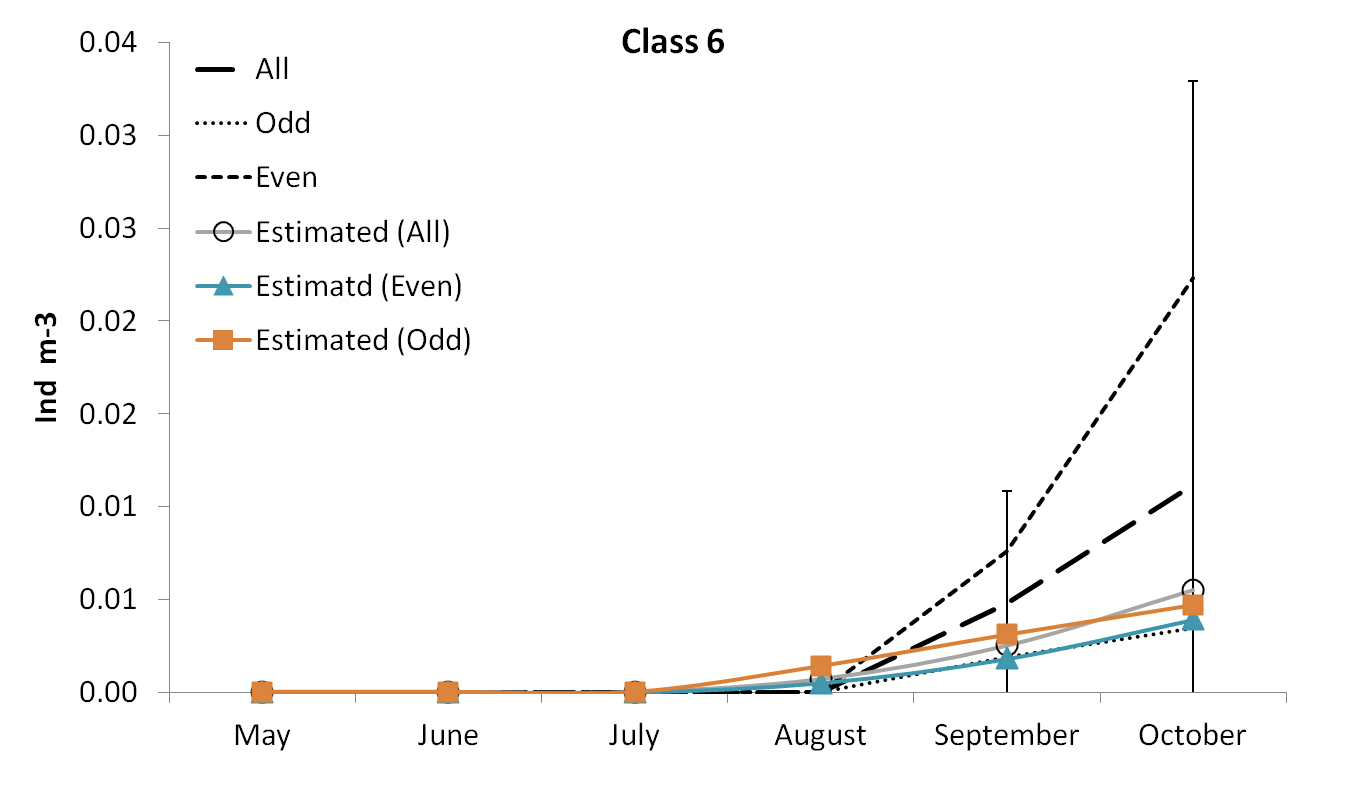


**Figure F. Class 6.** Black lines show monthly means for observed abundances from *all*, *odd*, and *even* data. Error intervals refer to 95% confidence intervals for *all* data. Solid color lines show the estimated abundances using *odd*, *even*, or *all* data.

REFERENCES

1. Kriegeskorte N, Simmons WK, Bellgowan PSF, Baker CI. Nature Neuroscience. 2009; 12:535-540.
